# 

**Published:** 1860-10

**Authors:** 


### INDEX.—VOL. XI.

PAGE.

A.

Artificial Dentures, . . . 16
Anchylosis of the Jaws, &c. 226
Antimony in Gold, . . . 298
Arctic Expedition, . . . 302
Anæsthesia and Anæsthetics, 363
Appeal of the Widow of Horace Wells, . . . . . 389
Asphyxia from Chloroform . 435
Arsenie Poisoning and Arsenic Eating, . . . . . 448
Apology, . . . . 454
Allen, Dr. J., Artificial Dentures, 16
Arsenic Eating, . . . . 513
Acupressure for the Arrest of Hemorrhage, . . . 561
Anæsthetic Electricity as an, . 594
Axillæ, Milk Secreted by, . 569
Arsenical Mineral Waters, . 581
Anatomy of Fifth Pair of Nerves, 586
Adulterations, . . . 592
Appointments, Professional, . 593

B.

Barbour, J. H., M. D., Diptherite, 382
Bernard, Claud. Study of the Parotid Gland . . . 414
Bread, Aerated, . . . 428
Burnham, H. B., M. D. Salivary Calculus, . . . 437
Base, Vulcanite, . . . 470
Bad Breath, . . . . 596
Brodie, Sir Benjamin, . . 596
Baillie, Dr. . . . . 596
Baltimore College, Change in Faculty, . . . . . 598

C.

Case, . . . . . 26
Colburn, G. F. J. Reproduction, &c., . . . . . 62
Chloroform, . . . . 69

PAGE.

Cancer, Extirpation of the entire Lower Lip, . . . 133
Case of Adipocere, . . . 142
Crania, . . . . 147
Contributors, . . . . 148
Casting Metallic Dies Direct, &c., 183
Clark, F. Y., D. D. S. Making Dies, . . . . 234
Corrections, . . . . 242
Cavities in Filling Teeth, . 325
Cartwright's Inaugural Address, 334
Chloroform Faradization, &c., 435
Currents and Counter Currents in Medical Science, . . . 447
Creasote, . . . . 481
Cavities, Filling of, . . . 510
Chloroform, on the Nature of Death from, . . . 551
Chloroform. Death from, . 555
Chloroform Liniment, . . 595
Ceratitis, . . . . 595
Cancer, Death from Inoculation, 598
Courts, Power of, over Medical Witnesses, . . . . 598
Cholera, . . . . 598
Convention, Medical, . . 594

D.

Dental Anomalies, &c., . . 3
Dentures, Artificial, . . . 16
Decay, Superficial, &c., . . 28
Decay, Dental, . . . . 71
Dentistry, Practical, . . . 176
Dental Profession. Hints to, &c., 181
Dies, Improvement in Casting, 183
Dies, A New Method, &c., . 234
Debray, H., Platina and Metals, 193
Distressing Nervous Symptoms, &c., . . . . . 307
Differences in Gold Foil, . 322
Dental Surgery, School of, . 334
Diptherite, . . . . 382
Dietitic Value of Fermented and Aerated Bread, . . . 428

PAGE.
Danglish, J., M. D., . . 428
Dental Education . . 446
Dentine, Treatment of Sensitive 467
Dentistry, the Practical Utility of, 475
Dentistry, Artificial, and Patents, 489
Death, on the Nature of, from Chloroform, . . . 551
Dental Society, . . 596
Dentists and R. C. of S., . 598
Death from Inoculation of Virus, 598
Delay of Number, . . 598
Delirium Tremens, Treatment of, 583
Diploma, Revoking of, . . 597
Digitalis in Delirium Tremens, 583
Dislocation, Painless Reduction of, 592

E.

Erratum, . . . 148

Exsection of Superior Maxilla, 371

Evils of Tight Lacing, . . 412

Education, Dental, . . 446

Eating, Arsenic, . . 448

Education, Physical, . 487

Electricity as an Anæsthetic, 594

F.

Filling Teeth, . . . 28

Formation of Shells, Bones, &c., 59

Fumes of Phosphorus, &c., . 64

Forget, A. M., M. D. Dental Anomalies, . . 149

Forming Cavities, . . 325

Face, Reparation of, &c., . 330

Fœtid Perspiration, . . 404

Faradization in Asphyxia, . 435

Facial Neuralgia, . . 498

Fractures of Inferior Maxilla, 504

Filling Carious Cavities, . 510

Fang Filling, . . 597

Filling Fangs, . . . 597

Faculty Change of, . . 598

Fractures of Superior Maxilla, 577

Fifth Pair of Nerves, . 586

G.

Gorgas, Dr. F. Case, . . 26

Growth of the Incisors in Rodents, 30

Glands, Salivary, . . 212

Gold Containing Antimony, 298

Gold Foils, the Difference, &c. 322

Gland, Extirpation of Parotid, 539

Gangrene Treated by Kreasote, 584

Gutta Percha as a Permanent Stopping, . . . . 586

H.

Harvey, Dr., . . . 57
Hayward, G., M. D., . 124

PAGE.
Hints and Suggestions to the Dental Profession, . . 181
House, Dr. J. Carroll. Improved Dies, . . 183
Hypnotism, . . . 292
Harvey, Leon F., M. D., 312
Hints, Practical, . . 316
Hoopes, Wm. H., D. D. S., Reparation of, &c., . . 330
Hulme's Mr., Lectures, . 353
Hamilton Prof. F. H., Paralysis, 406
Hypochlorite of Lime, . 438
Hospital, Long Island, . . 406
Harriss, Dr. W. A., . 504
Hemorrhage, for the Arrest of, 561
Harris, Prof. C. A., Obituary of, 588
Homeopathic College, . 593
Hypnotism, . . . . 592

I.

Influence on the Production of Disease, . . . . 3
Incisors in Rodents, . . 30
Inaugural Address, . . 82
Injection Submucous, for the Cure of Tooth-ache, . . 138
Improved Method of Casting Dies, 183
Inaugural Address, . . 334
Interstitial Keratitis, . . 419
Impressions, Plaster, . 597
Indian Medical Journal, . 597

J.

Jaw Lower, Necrosis of, from, &c. 64
Jaws, Tumors of, &c., . 105
Jaws, Anchylosis of, &c., . 226
Jigger, the . . . 423
Journale de Chimie Medicale, . 454

K.

Keratitis, Interstitial, . . 419
Kreasote in Gangrene, . 584

L.

Letter from Dr. Harvey, . 57
Lip, Lower, Extirpation of, &c., 133
Longevity, . . . . 146
Lower Jaw, Anchylosis of, &c., 226
Levison, Dr. J., . . 307
Loosening of Plates, . 314
London School of, &c., . . 334
Lectures, Mr. Hulmes . 353
Liniment of Chloroform, 595

M.

McCalla, J., D. D. S., . 28

PAGE.
Metropolitan School of D. S., . 82
Maxilla, Resection of, &c., 133
Metals which Accompany Platina, 193
Maryland and Virginia Medical and Surgical Journal, . 303
Maxillary, Exsection of, &c., . 371
Movement Cure, . . 472
McElroy, Dr. C., Neuralgia, 498
Maxilla Fractures of Inferior, 504
Manna, of the Hebrews, . 562
Mallet, the . . . . 597
Milk, Secreted by Tumors in the Axillæ, . . . 569
Maxilla, Fractures of Superior, &c.577
Medical Convention, . . 594

N.

Necrosis of the Lower Jaw, &c. 64

Necrosis-Phosphor, . . 67

New Method for Dies, . 234

Newspaper Literature, . . 299

Necrology, . . . . 303

Nervous Symptoms in Smokers, 307

Naples and Salerno, . . 387

New Medical Journal, . 454

Neuralgia Facial, &c., . . 498

Needle for Sutures, . . 594

New Disease of the Gums, . 592

O.

On Filling Teeth, . . 28
On Continued Growth of the Incisors, &c., . . . . 30
On the Formation of Shells and Bone, . . . 59
On Dental Decay, . . 71
Our Contributors, . . 148
On Tic Douloureux, . . 254
On the Action of Hypochlorite, 438
Oudet, J. E., . . . 30
Organic Matter, Influence of Sun's Rays upon, . . . 557
Orum & Armstrong's Teeth, 593

P.

Phosphor Necrosis, . . 67
Phosphorous, Fumes of, &c., . 64
Pregnancy, Tooth-ache in, &c. 138
Practical Dentistry, . . 176
Plaster Impressions, . . 183
Profession in England, . 250
Platina and its Metals, 301, 514
Plates, Loosening of, . . 314
Practical Hints, . . 316
Palate, Reparation of, . . 330
Professional Pecksniffs, . 410
Parotid Gland, . . . 414
Proceedings of the Massachusetts Medical Society, . . 439

PAGE.
Physical Education, . . 487
Patents and Artificial Dentistry, 489
Parotid Gland, Extirpation of, 539
Psychology, . . . 595
Plaster Impressions, . . 597
Platinum, . . . 596
Pivot Teeth, . . . 596

Q.

Quackery, and who are, &c., 20

R.

Reproduction of Canine Teeth, 62
Richardson, Dr., Address, . 82
Report on Tumors of the Jaws, 105
Remarks on Anæsthesia, . 124
Resection of, &c., . . 133
Remarkable Case of Adipocere, 142
Reparation of the Face, and Palate, 330
Revoking a Diploma, . 597
R. C. of S. Dentists, and the, 598

S.

Societies, Dental, . . 42
Shells, Formation of, &c. . 59
Submucous Injection, . . 138
Salivary Glands, . . 212
Surgery, Tomes, . . 148
Shepherd, S. M., on Patents, 489
Sutures, Novel Needle for, . 594
Scrofula, . . . . 595
Surgery, Principles and Practice of, . . . . 586

T.

Thomson, F. H., M. D., . 71
Tumors, Report on, &c., . 105
Tooth-ache of Pregnancy, . 138
Tomes' Dental Surgery, . 148
Taft's Operative Dentistry, . 148
Thompson, T. D., D. D. S., 181
Tobacco—its Effects upon the Health and Teeth, . . 455
Treatment of Sensitive Dentine, 467
Trephine, a new, . . 596
Teeth, Pivot, . . 596
The Mallet, . . . 597
Teeth. Orum & Armstrong's, 593

V.

Veratrine—its Use in Dental Surgery, . . . . 312
Vulcanite Base, . . . 470

W.

Witnesses, Power of Courts over, 598

